# Disseminated cryptococcosis with varicella-zoster virus coinfection of idiopathic CD4 + T lymphocytopenia: a case report and literature review

**DOI:** 10.1186/s12985-022-01765-7

**Published:** 2022-03-05

**Authors:** Li Fang, Junli Zhang, Fangfang Lv

**Affiliations:** Department of Infectious Disease, Sir Run Run Shaw Hospital, School of Medicine, Zhejiang University, 3 Qingchun East Road, Hangzhou, 310020 China

**Keywords:** Cryptococcosis, Cryptococcal meningitis, Varicella-zoster virus, Idiopathic CD4 + T lymphocytopenia

## Abstract

**Background:**

Idiopathic CD4 + T lymphocytopenia (ICL) is a rare immunodeficiency syndrome, unaccompanied by various opportunistic infections. Cryptococcus and varicella-zoster viruse are the most common opportunistic infections.

**Method:**

We described a case of disseminated cryptococcosis with varicella-zoster virus coinfection in a patient with ICL and reviewed all published reports. A total of 26 cases with cryptococcal meningitis in ICL were enrolled.

**Discussion:**

ICL remains poorly understood to clinicians. Patients with cryptococcal meningitis in ICL mostly suffered with headache and fever in a subacute or chronic period, while some patients might have atypical manifestations which makes a difficulty for early diagnosis. Some characteristics of cerebrospinal fluid can help to predict the prognosis of the disease. Cryptococcosis with varicella-zoster virus coinfection is rare but serious.

**Conclusion:**

We recommed CD4 + T cells should be assessed in patients with unusual or recurrent infections. As the underlying pathophysiology is poorly understood, there is no standard therapy for ICL. Increased awareness of the disease and early prevention for CD4 reduction are needed.

## Background

Idiopathic CD4 + T lymphocytopenia (ICL) is a rare immunodeficiency syndrome with an unexplained reduction of CD4 + T lymphocytes and no evidence of Human Immunodeficiency Virus (HIV) infection or any other cause of immunodeficiency [1]. It is defined as a documented absolute CD4 + T-cell count < 300 cells/L or < 20% of total lymphocytes on at least two occasions, usually two or three months apart [2]. Patients with ICL typically present with opportunistic infections, malignancies, or autoimmune disorders. Cryptococcus infection is the most common opportunistic infection in ICL patients [1]. Cryptococcal meningitis is the most serious disease with high morbidity and mortality [3–5]. Here, we present a case of disseminated cryptococcosis with varicella-zoster virus coinfection in a patient with ICL, and the relevant literature is reviewed. ICL should not be ignored in some patients who seem to be immunocompetent.

## Case presentation

A 44-year-old man was admitted to the emergency department with a complaint of headache lasting for 10 days without fever or any other symptoms, and he was prescribed some painkillers. He suffered fever, vomiting, delirious and urinary retention two weeks later. He had no medical or surgical history and no history of drug or alcohol abuse. Vital signs included a body temperature of 38.5 °C, respiration of 20 breaths/min, heart rate at 97 beats/min, blood pressure of 133/90 mmHg, and O_2_ saturation of 97% on ambient air. Physical examination showed a decreased muscle strength of both lower limbs and neck was stiff with positive Kernig's and Brudzinki's signs.

**Fig. 1:**
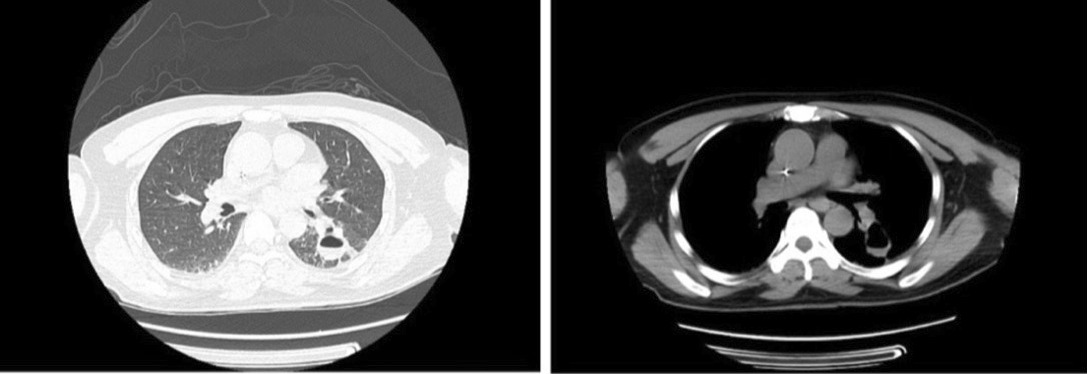
Lung CT scan revealed cavernous lesions in the lower lobe of the left lung and a few fibrous foci in the right lung's middle lobe, as well as a minor amount of pleural effusion

Laboratory tests results are as follows: a leukocyte count of 15.3/mm^3^ (reference range 3.5–9.5/mm^3^), with neutrophils 14.18/mm^3^ (reference range 1.8–6.3/mm^3^) and lymphocytes 0.5/mm^3^ (reference range 1.1–3.2/mm^3^), high-sensitive c-reactive protein 26.5 mg/L (reference range 0–5 mg/L), Erythrocyte Sedimentation Rate 46 mm/h (reference range 0–20 mm/h), potassium 2.95 mM/L (reference range 3.5–5.5 mM/L), sodium 132 mM/L (reference range 137–147 mM/L), chlorine 92 mM/L (reference range 99–110 mM/L), and serum creatinine 163 μM/L(reference range 40–106 μM/L). The biochemistry testing on the liver was normal. A lumbar puncture (LP) showed an elevated open pressure of > 40 cmH_2_O (1 cmH_2_O = 0.1 kPa). Cerebrospinal fluid (CSF) analysis revealed 90 /mm^3^ leukocyte (reference range 0–5/mm^3^), with 9% neutrophils and 86% lymphocytes. The glucose level was 0.28 mM/L (reference range 2.5–4.44 mM/L), chloride level was 116 mM/L (reference range 119–129 mM/L) and protein level was 0.898 g/L (reference range 0.15–0.45 g/L). Polymerase chain reaction (PCR) for Herpes simplex, Epstein Barr virus, Varicella, Cytomegalovirus were negative. Acid-fast staining was negative, India ink preparation was positive, and cryptococcal antigen showed positive at 76.6 μg/L. The mycological culture was positive for *Cryptococcus neoformans* variant. A head computed tomography (CT) scan revealed no signs of hydrocephalus. The electroencephalogram showed a minor anomaly, while a lung CT scan revealed cavernous lesions in the lower lobe of the left lung, and pleural effusion was observed bilaterally, as well as a few fibrous foci in the right lung's middle lobe (Fig. 1).

A thorough analysis to rule out immunocompromised status was performed. The HIV serology and HIV RNA, T-SPOT.TB tests were negative, the antinuclear antibody was negative, and the serum immunoglobulins and complement were normal. The anti-IFN-γ autoantibodies linked to disseminated nontuberculous mycobacterial infections were normal. He had serially decreased lymphocytes during his follow-up, particularly CD4 + T cells in Table 1.

**Table 1:** Blood cell counts of the patient with CD4 + T lymphocytopenia

Date of analysis	Count, /mm^3^					
	white blood cells 3.5–9.5	Lymphocytes 1.1–3.2	CD3 + CD4 + cells 0.20–1.82	CD3 + CD8 + cells 0.13–1.35	CD16/56 + cells^*^ 0.04–1.00	CD19 + cells^*^ 0.05–0.67
March 27	9.6	0.4	0.04	0.18	0.02	0.17
April 6	7.7	0.5	0.06	0.26	0.01	0.1
April 26	4.4	1.0	0.12	0.71	0.03	0.04
June 29	6.5	1.5	0.18	0.84	0.04	0.28

CD16/56 + cells^*^: natural killer cells, CD19 + cells^*^: B cells

**Fig. 2:**
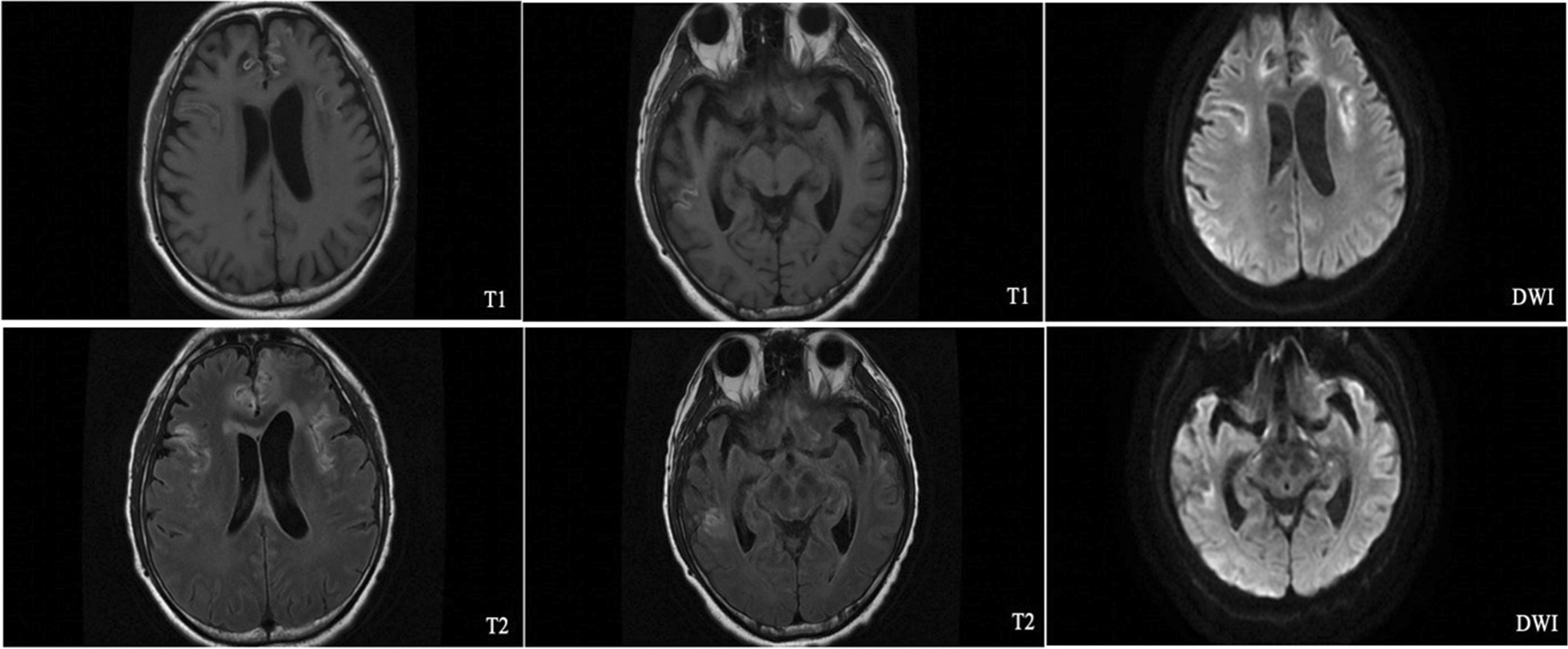
Brain MRI scan revealed multiple lesions and lamellar necrosis in the parietal lobe of the frontotemporal island and the cortex of the left cerebellar hemisphere, also we observed hydrocephalus in several lesions, accompanied by interstitial edema

The patient was treated with amphotericin B (0.7 mg/kg daily) and 5-fluorocytosine (2.5 g per 12 h), an Ommaya reservoir was implanted for cerebrospinal fluid drainage. He was deteriorated in the second week of hospitalization, with persistent positive CSF culture and a higher CSF cryptococcal antigen result (3097.6 μg/L). During the fourth week of hospitalization, zonal dispersed papules were seen on the right lower leg of the patient, with reduced muscular strength in both lower limbs, and he was suffered from hallucinations and auditory hallucinations. Cerebrospinal fluid was hemorrhagic, with 6 /mm^3^ leukocyte and 700 /mm^3^ erythrocyte. Magnetic resonance imaging (MRI) showed multiple lesions and lamellar necrosis in the parietal lobe of the frontotemporal island and the cortex of the left cerebellar hemisphere. We also observed hydrocephalus in several lesions, accompanied by interstitial edema (Fig. 2). Varicella-zoster virus and cryptococcus were positive in metagenomic next-generation sequencing (mNGS) of cerebrospinal fluid, and ganciclovir was started for antiviral therapy. Unfortunately, he finally died of cerebral edema and respiratory failure after 3 months of admission.

## Literature review and discussion

ICL is a rare condition that is found worldwide, with unknown incidence and etiology, and it is viewed as a syndrome that likely encompasses different disorders caused by the reduction of CD4 cell numbers. CD4 is a glycoprotein expressed on the surface of various types of helper and regulatory T cells. CD4 + T cell is an important immune cell that regulates the activities of cells participating in immune responses, participate in apoptosis (programmed cell death), and tumor monitoring. Patients with ICL will develop opportunistic infections, malignancies, and/or autoimmune diseases [6]. Cryptococcal meningitis is the most common opportunistic infection. According to a series of studies, one-third of patients were infected with cryptococcus, while around 10% were infected with the varicella-zoster virus [7]. Cryptococcus was reported to be the most frequent infection in 258 ICL patients (26.6%), followed by mycobacterial (17.0%), candidal (16.2%), and varicella-zoster virus infections (13.1%) [8].

CD4 + T cells should be assessed when the patient presents with unusual or recurrent infections. However, the infection can be associated with lymphocyte changes, and it is impossible to determine whether changes reflect a primary immunologic defect or the response to infection. Our patient suffered disseminated cryptococcal infection and herpes virus infection with no underlying disease and HIV or tuberculosis, but a series of low CD4 + T cells lasting for more than 3 months. As reported, anti-cytokine autoantibodies have also been related to opportunistic infections, with anti-IFNγ autoantibodies linked to disseminated nontuberculous mycobacterial infections and anti-IL-17 autoantibodies linked to chronic mucocutaneous candidiasis, and anti-IL-6 autoantibodies linked to Staphylococcal infections [9]. Anti-GM-CSF autoantibodies have also been found in a few cryptococcal meningitis patients caused by *Cryptococcus*. *Gattii,* but not *Cryptococcus*. *neoformans* [10, 11]. However, CD4 + T cells were normal or slightly lower in most patients [12].

Cryptococcal meningitis is not uncommon in the clinic, and ICL remained poorly understood to clinicians. There are few systematic reviews on ICL and cryptococcal meningitis. A systematic search was performed on PubMed between 1992 and December 2020. A combination of the following search terms was used: cryptococcosis, cryptococcus infection, cryptococcal meningitis, idiopathic CD4 lymphocytopenia, ICL, HIV negative CD4 lymphocytopenia. 26 cases were enrolled for analysis [13–36]. Among those patients, 20 (76.9%) were male, 6 (23.1%) were female. The median age was 42 (range 4.5–75) at diagnosis. Cryptococcosis in ICL patients usually had a subacute or chronic course and took weeks to months from symptom until diagnosis [37]. The most common symptoms were headache, fever, nausea/vomiting, and meningeal irritation. The symptoms in those 26 patients were described in Table 2. The primary symptoms were headache and fever (73.1%, 61.5%). Nausea, vomiting, and disorientation were also common. The patients suffered only headaches at the early period. Therefore, some patients might have atypical manifestations during the process of disease.

**Table 2:** Presenting Symptoms of cryptococcal meningitis in ICL

Symptoms	No. of patients (%)
Headache	19 (73.10)
Fever	16 (61.50)
Nausea/vomiting	9 (34.60)
Confusion	8 (30.80)
Dizziness	4 (15.40)
Neck pain	3 (11.50)
Hemiparesis	3 (11.50)
Ataxia	3 (11.50)
Weight loss	3 (11.50)
Diplopia	3 (11.50)
Experiencing speech difficulties	2 (7.70)

Cerebrospinal fluid were analysed in 22 cases with adequate information in Table 3 [13–19, 21–24, 26, 27, 29, 31–36]. It showed a median leukocyte of 61 cells/μL (ranging 0–700), mainly constituted by lymphocytic, glucose of 39.1 mg/dL (ranging 1.98–87 mg/dL), below 40 mg/dL (1 mM/L = 18 mg/dL) in 50% of the patients, and protein of 116 mg/dL (ranging 20–266 mg/dL). The India ink stain and cryptococcal antigen titer positive rates were 12/14 (86%) and 15/16 (94%), respectively. The positive rate of CSF culture was 18 /19 (95%), Only one case was *Cryptococcus*. *gattii* and all the others were *Cryptococcus neoformans.* Cryptococcal meningitis is linked to a high rate of morbidity and death. Poor outcomes have previously been linked to advanced age (≥ 60 years), solid malignancy, hematologic malignancy, liver cirrhosis, respiratory failure, long-term ICU stay, corticosteroid treatment, and disturbed mental state (coma, seizure, herniation) [38–41]. Low CSF leukocyte counts (less than 20 cells/microL), low CSF glucose, high CSF CrAg titers (> 1:1024), high CSF opening pressure (≥ 250 mm H_2_O), lower Glasgow Coma Scale (GCS) scores, hematogenous dissemination of cryptococcosis, hydrocephalus, and cerebral infarction have all been linked to poor outcomes [38, 40–46].

**Table 3:** Presenting characteristics in cerebrospinal fluid of cryptococcal meningitis in ICL patients

References	Protein (mg/dL)	Glucose (mg/dL)	Leukocyte (cells/mm^3^)	Predominant cell	India ink stain	Cryptococcal antigen	Culture
Sim et al. [13]	266	1.98	10	NA	Positive	1:2560	*Cryptococcus neoformans*
Eshwara et al. [14]	50	60	54	Lymphocyte	Positive	Negative	*Cryptococcus gattii*
Malone et al. [15]	48	87	45	Lymphocyte	Positive	1:512	*Cryptococcus neoformans*
Shribman et al. [17]	83	34.2	NA	Lymphocyte	NA	1:1280	*Cryptococcus neoformans*
Shribman et al. [17]	89	19.8	212	Lymphocyte	NA	Positive	*Cryptococcus neoformans*
Ivica et al. [18]	138	66.6	478	Neutrophils	Positive	NA	*Cryptococcus neoformans*
Sancesario et al. [19]	133	23	23	NA	Negaitive	NA	NA
Sharma et al. [21]	125	17	0	0	NA	1:8192	*Cryptococcus neoformans*
Augustine et al. [22]	107	31	125	Lymphocyte	Negative	NA	negative
Augusto et al. [23]	60	28.11	43	Lymphocyte	Positive	NA	*Cryptococcus neoformans*
Yilmaz-Demirdag et al. [24]	175	44	75	Neutrophils	Positive	1:1024	*Cryptococcus neoformans*
Jha et al. [26]	80	NA	NA	NA	Positive	Positive	NA
Salit et al. [27]	227	65.88	126	NA	NA	1:2048	*Cryptococcus neoformans*
Lepur et al. [39]	191.5	3.6	700	Lymphocyte	Positive	NA	*Cryptococcus neoformans*
Lepur et al. [29]	155.5	10.8	450	Neutrophils	NA	NA	*Cryptococcus neoformans*
Cheung et al. [31]	NA	NA	13	Neutrophils	Positive	1:4.56	*Cryptococcus neoformans*
C.L.HO et al. [32]	NA	48	0	0	NA	1:64	NA
Yinnon et al. [33]	NA	NA	87	NA	NA	1:1024	*Cryptococcus neoformans*
Ramirez et al. [34]	20	68	0	0	NA	1:1024	*Cryptococcus neoformans*
Ostrowski et al. [35]	NA	NA	98	Lymphocyte	Positive	1:8192	*Cryptococcus neoformans*
Duncan et al. [36]	25	54	0	0	Positive	1:8192	*Cryptococcus neoformans*
Duncan et al. [36]	172	48.96	68	Lymphocyte	Positive	1:16,384	*Cryptococcus neoformans*

Cryptococcal infection and ICL have an increased likelihood of developing dermatomal zoster [47]. However, due to a lack of systems analysis, it is not sure whether this coinfection can be more severe than a single infection. We consider the deterioration of our patient is related to the activation of the varicella-zoster virus. We believe it may also require more cases or literature reviews.

## Conclusion

Low CD4 + T cell counts characterize idiopathic CD4 + T lymphocytopenia and commonly presents as various opportunistic infections, autoimmune diseases, and/or neoplasias. Patients with opportunistic infections with HIV negative should be evaluated for ICL. Here we present a case of disseminated cryptococcosis with varicella-zoster virus coinfection, with an adverse outcome. As the underlying pathophysiology is poorly understood, there is no standard therapy for ICL. The focus is still on the preventive treatment of CD4 + T cell reduction.

## Data Availability

All data generated or analyzed during this study are included in this published article.

## Abbreviations

ICL: Idiopathic CD4 + T lymphocytopenia
HIV: Human immunodeficiency virus
CSF: Cerebrospinal fluid
PCR: Polymerase chain reaction
CT: Computed tomography
MRI: Magnetic resonance imaging
mNGS: Metagenomic next-generation sequencing
GCS: Glasgow Coma Scale
